# Effectiveness and safety profile of cladribine in an Italian real-life cohort of relapsing–remitting multiple sclerosis patients: a monocentric longitudinal observational study

**DOI:** 10.1007/s00415-023-11700-7

**Published:** 2023-04-07

**Authors:** Chiara Zanetta, Maria A. Rocca, Alessandro Meani, Vittorio Martinelli, Laura Ferrè, Lucia Moiola, Massimo Filippi

**Affiliations:** 1 Neurology Unit, IRCCS San Raffaele Scientific Institute, Via Olgettina, 60, 20132 Milan, Italy; 2 Neurorehabilitation Unit, IRCCS San Raffaele Scientific Institute, Milan, Italy; 3 Neuroimaging Research Unit, Division of Neuroscience, IRCCS San Raffaele Scientific Institute, Milan, Italy; 4 Vita-Salute San Raffaele University, Milan, Italy; 5 Neurophysiology Service, IRCCS San Raffaele Scientific Institute, Milan, Italy

**Keywords:** Relapsing–remitting multiple sclerosis, Cladribine tablets, Real-world data, Observational study, Effectiveness, Safety

## Abstract

**Introduction:**

Cladribine is approved for the treatment of active relapsing MS (RRMS), but its positioning in MS therapeutic scenario still needs to be fully elucidated.

**Methods:**

This is a monocentric, observational, real-world study on RRMS patients treated with cladribine. Relapses, magnetic resonance imaging (MRI) activity, disability worsening, and loss of no-evidence-of-disease-activity-3 (NEDA-3) status were assessed as outcomes. White blood cell, lymphocyte counts and side effects were also evaluated. Patients were analyzed overall and in subgroups according to the last treatment before cladribine. The relationship between baseline characteristics and outcomes was tested to identify predictors of response.

**Results:**

Among the 114 patients included, 74.9% were NEDA-3 at 24 months. We observed a reduction of relapses and MRI activity, along with a stabilization of disability. A higher number of gadolinium-enhancing lesions at baseline was the only risk factor for loss of NEDA-3 during follow-up. Cladribine was more efficacious in switchers from first-line therapies or naïves. Grade I lymphopenia was more frequent at month 3 and 15. No grade IV lymphopenia cases were observed. Independent predictors of grade III lymphopenia were a lower baseline lymphocyte count and a higher number of previous treatments. Sixty-two patients presented at least one side effect and globally 111 adverse events were recorded, none of them was serious.

**Conclusions:**

Our study confirms previous data on cladribine effectiveness and safety. Cladribine is more effective when placed early in the treatment algorithm. Real-world data on larger populations with longer follow-up are needed to confirm our findings.

## Introduction

Multiple sclerosis (MS) is a chronic disorder of the central nervous system (CNS) characterized by inflammation and neurodegeneration. MS is a multifactorial disease, which involves a combination of genetic, environmental and immunological factors. Aberrant targeting of the CNS by the immune system plays a central role in the pathogenesis, causing inflammation and damage to myelin and myelin-producing cells [1]. The therapeutic scenario for patients affected by MS has enormously increased in recent years, with the availability of various agents with different mechanisms of action. These treatments include cladribine (CLAD), which is a deoxyadenosine synthetic analog prodrug that induces a transient lymphocyte apoptosis and depletion, with only a minimal effect on the innate immune system, followed by an immune reconstitution with improved immune tolerance [2]. CLAD is administered with a short treatment course, thus offering the advantages of a few treatment days per year (8–10 days annually). Despite this, its mode of action is believed to be responsible for a durable clinical effect [2].

CLAD has been approved for the treatment of active relapsing MS (RRMS) as defined by clinical or magnetic resonance imaging (MRI) features [3]. However, its positioning in the MS therapeutic landscape and predictors of response to treatment still need to be fully elucidated.

In the Cladribine Tablets Treating Multiple Sclerosis Orally (CLARITY) (ClinicalTrials.gov, NCT00213135) and CLARITY Extension studies (ClinicalTrials.gov, NCT00641537), which investigated safety and efficacy profile of CLAD, patients with active RRMS were randomized to placebo or a cumulative dose of CLAD tablets 3.5 mg/kg body weight for 2 years. The results showed an improvement in relapse rate, disability progression and MRI measures of disease activity, along with a good safety profile. The most commonly reported adverse event (AE) was mild to moderate lymphopenia, as expected by the mechanism of action [4]. Real-world evidence from the Italian MS Registry, evaluating CLAD long-term effectiveness of subjects previously treated in the context of the trials, is in line with data from CLARITY and CLARITY Extension studies, showing that half of the MS subjects were free from relapses and disability progression over 5 years of observation [5].

Although data from clinical trials demonstrated that CLAD is effective and well tolerated in patients with MS, with a low burden of monitoring during and following treatment, little is known about its performance in a real-world setting and available data coming from the real-life need to be confirmed and validated with further evidence. Furthermore, predictors of response to CLAD and factors influencing its effectiveness are still not fully elucidated.

Here, we report real-world findings about effectiveness and tolerability of CLAD in a monocentric Italian cohort of RRMS patients, identifying early predictors of response and risk factors for suboptimal response to treatment.

## Methods

This is a monocentric, observational, real-world study. Data from adult patients with MS based on 2017 revised McDonald Criteria [6] who started CLAD treatment according to clinical practice from April 2018 to November 2021 were both retrospectively and prospectively collected at San Raffaele Hospital MS Center. Demographic, clinical and MRI data were examined.

Both patients with MS who started CLAD as first treatment (naïves) and subjects who were previously treated with different disease-modifying treatments (DMTs) (switchers) were considered. We included subjects with a minimum of 6 months of follow-up since CLAD first administration. Patients who had not yet received CLAD retreatment were also included. The 6-month on-treatment persistence was established to exclude rebound disease activity in switchers from other DMTs and to be consistent with data from clinical trials showing improvement in clinical and MRI outcomes 24 weeks after CLAD first administration [4, 7].

At baseline, which means at CLAD start, we collected the following variables: sex, date of birth, date of MS diagnosis, date of CLAD start, age at CLAD start, disease duration before CLAD start, Expanded Disability Status Scale (EDSS), annualized relapse rate (ARR) 1 and 2 years before CLAD start, number and type of previous DMTs, reasons for previous DMTs discontinuation, number of T2-hyperintense brain lesions, presence and number of brain gadolinium-enhancing lesions at the most recent available MRI performed before CLAD start.

CLAD was administered according to national prescribing criteria (3.5 mg/kg body weight over 2 years, one treatment course per year, each treatment course consisting of 2 treatment weeks). Patients with MS were evaluated every 3 months with a standardized neurological examination, including EDSS rating, collection of relapses and adverse/side effects, and routine blood tests, including white blood cell (WBCs) and lymphocyte counts (ALCs) and liver enzyme values. Lymphopenia severity grades were categorized according to the Common Terminology Criteria for Adverse Events (CTCAE) v5.0 [8]. Relapses and AEs were also evaluated at unscheduled visits, if necessary.

MRI examinations performed as per clinical practice were also collected and reviewed. A re-baseline MRI was performed 4–6 months after CLAD first course and subsequent scans were scheduled yearly or at different time points according to clinical practice.

Follow-up data were recorded from baseline to the last available visit up to 15th September 2022. The following efficacy variables were collected: date of CLAD retreatment, presence and date of clinical relapses, EDSS, presence and number of new/enlarged T2-hyperintense and/or gadolinium-enhancing lesions.

“No evidence of disease activity-3” (NEDA-3) status, intended as absence of clinical relapses, disability progression and active MRI lesions, was evaluated during follow-up after CLAD start. A relapse was defined as any new neurological symptom lasting for at least 24 h, not associated with fever or infection, and accompanied by new neurological signs [6]. MRI activity was defined as the presence of new/enlarged T2-hyperintense and/or gadolinium-enhancing lesions compared to previous MRI. Worsening of disability was established if an increase in the EDSS was confirmed at 2 independent clinical assessments 6 months apart as follows: + 1.5 points (if baseline EDSS = 0.0), + 1.0 point (if baseline EDSS = 1.0–5.5) and + 0.5 points (if baseline EDSS ≥ 6.0).

Switcher patients were stratified according to last DMT before CLAD start. First-line agents included: interferons, glatiramer acetate, teriflunomide and dimethyl fumarate. Second-line drugs included fingolimod, natalizumab and ocrelizumab.

Estimates of the proportion of patients without clinical relapses, confirmed disability worsening, MRI activity, and loss of NEDA-3 status were obtained using the product-limit approach. Kaplan–Meier curves were compared among the groups studied by log-rank test. Cox regression models were run to screen baseline demographic, clinical and MRI features as potential risk factors for suboptimal response to CLAD. Candidate predictors (*p* < 0.1, uncorrected, at univariable analysis) entered the multivariable analysis, testing both forward and backward stepwise selection algorithms, based on Schwarz Bayesian Information Criterion (BIC). To assess the stability of selected subsets, we ran LASSO regularized Cox models, regressing each outcome on the whole set of examined features. The optimal value of the tuning parameter, which controls the amount of penalty and promotes variable selection, was chosen according to the within-1-standard-error rule from the minimum partial likelihood reached in a tenfold cross-validated (CV) scheme.

We assessed and compared ARR per epoch using negative binomial generalized estimating equations (GEE) for longitudinal data. Similar models were run to evaluate the number of new/enlarged T2 and gadolinium-enhancing lesions. We studied EDSS score trend over time with linear mixed models. All analyses were performed in the whole cohort, and according to last DMT before CLAD start including in each model specific interaction terms.

WBCs and ALCs over time in the whole cohort, as well in naïves and switchers from first and second lines, were assessed with linear mixed models. We repeated the analysis evaluating separately patients switching from dimethyl fumarate and fingolimod, which are known to potentially induce lymphopenia. The distribution of lymphopenia severity grade over time was obtained, with a focus at month 3, month 15 and patients’ nadir. Frequencies of occurrence of other AEs were reported. We looked at baseline predictors of grade III/IV lymphopenia over time and tested the association of grade III/IV lymphopenia with the risk of infections by GEE logistic regression models.

SAS release 9.4 (SAS Institute, Cary, NC) and R (version 4.2.2) software were used for computations.

## Results

### Population characteristics

Table 1 shows baseline characteristics of our cohort, overall and stratified according to last DMT.

**Table 1:** Demographic, clinical and MRI characteristics of multiple sclerosis patients starting cladribine at baseline

	All	Naives	1st-lines	2nd-lines	*p*
Total *N* (Female *N*; %)	**114** (**82**; 71.9)	**57** (**42**, 73.7)	**35** (**21**, 60.0)	**22** (**19**, 86.4)	0.102^a^
Age at CLAD start (y), mean (SD)	**33.0** (9.2)	**32.4** (9.9)	**33.3** (9.3)	**34.1** (7.1)	0.747^b^
MS duration pre CLAD start (y), median (IQR)	**3.0** (0.7–8.3)	**0.7** (0.3–1.5)	**7.2** (3.1–11.7)	**8.3** (5.1–12.5)	**< 0.001**^c^
EDSS at CLAD start, median (IQR)	**2.0** (1.5–2.5)	**2.0** (1.5–2.5)	**1.5** (1.0–2.5)	**1.5** (1.0–2.5)	0.159^c^
1 year before ARR, mean (SD)	**1.3** (0.8)	**1.6** (0.6)	**1.1** (0.8)	**1.0** (0.9)	**< 0.001**^c^
2 years before ARR, mean (SD)	**1.2** (0.8)	**1.3** (0.6)	**1.0** (0.7)	**1.3** (1.1)	0.068^c^
Treatment characteristics					
*N* Previous treatments, median (IQR)	**0.5** (0.0–2.0)	**–**	**1.0** (1.0–2.0)	**2.0** (1.0–3.0)	
Category of *N* Previous treatments, *N* (%) 0 1 2 > 2 Type of Last Previous treatments, *N* (%) Interferon Glatiramer acetate Teriflunomide Dimethyl fumarate Fingolimod Natalizumab Ocrelizumab	**57** (50.0) **26** (22.8) **15** (13.2) **16** (14.0) **4.0** (3.5) **9.0** (7.9) **5.0** (4.4) **17** (14.9) **17** (14.9) **4** (3.5) **1** (0.9)	**57** (100.0) – – – – – – – – – –	– **18** (51.4) **10** (28.6) **7** (20.0) **4.0** (11.4) **9.0** (25.7) **5.0** (14.3) **17** (48.6) – – –	– **8** (36.4) **5** (22.7) **9** (40.9) – – – – **17** (77.3) **4** (18.2) **1** (4.5)	**< 0.001**^**c**^
Brain T2-weighted lesion count, median (IQR)	**16.0** (10.0–30.0)	**16.0** (10.0–33.5)	**13.5** (10.0–25.0)	**22.0** (10.0–31.0)	0.538^c^
Brain gadolinium-enhancing lesion count, median (IQR)	**1.0** (0.0–3.0)	**2.0** (1.0–3.0)	**1.0** (0.0–2.0)	**1.0** (0.0–1.5)	**0.019**^**c**^
Follow-up post CLAD start (m), median (IQR)	**25.2** (14.6–38.7)	**24.0** (14.0–39.6)	**22.9** (16.2–34.3)	**30.9** (14.6–43.5)	0.558^c^

N = number of subjects included in the study; CLAD = cladribine; y = years; SD = standard deviation; IQR = interquartile range; MS = Multiple Sclerosis; EDSS = Expanded Disability Status Scale; ARR = Annualized Relapse Rate; m = months

^a^Fisher’s exact test; ^b^linear models; ^c^Kruskal–Wallis Test

Our study included 114 patients with MS treated with CLAD with a minimum follow-up of 6 months; 82 were female (71.9%).

Disease duration was significantly shorter in naïves compared to patients switching from first- and second-line agents (*p* < *0.001*), with no differences between the two categories of switchers (*p* = *0.585*).

ARR 1 year before CLAD start was higher in naïves (*p* ≤ *0.005*), with no difference between switchers from first- and second-line agents (*p* = *0.845*). Similarly, numbers of previous treatments before CLAD did not differ between switchers (*p* = *0.181*).

Four subjects switched to CLAD from interferon, 9 from glatiramer acetate, 5 from teriflunomide, 17 from dimethyl fumarate, 17 from fingolimod, 4 from natalizumab and 1 from ocrelizumab. Reasons for switching were inefficacy (*n* = 53, 93.0%), safety (*n* = 2, 3.5%) and pregnancy planning (*n* = 2, 3.5%).

The number of brain gadolinium-enhancing lesions at baseline was higher in naïves (*p* ≤ *0.039*), with no differences between switchers (*p* = *0.449*).

Duration of follow-up post CLAD start was not significantly different in the three groups of patients (*p* = *0.558*).

A total of 89 patients (78%) received CLAD retreatment. The recommended treatment interval between first and second course was usually maintained with the exception of 4 patients: 3 patients postponed retreatment for persistent lymphopenia and 1 for active *Helicobacter Pylori* infection.

### Cladribine effectiveness profile

#### Disease activity in the first 6 months

Seven patients (6.1%) presented clinical relapses in the first 6 months since CLAD start: 2 subjects were naïves, 2 switched from first-line therapies and 3 from second-line treatments. Of them, only 1 patient, switching from fingolimod, presented further clinical disease activity during follow-up.

Thirty-six patients (31.6%) experienced MRI activity in the first 6 months since CLAD start: 16 naïves, 11 switchers from first-line DMTs and 9 from second-line. Median new/enlarged T2 and gadolinium-enhancing lesion numbers in the first 6 months were 1 and 0, respectively. Eight (22.2%) of these patients (4 naïves, 1 and 3 switchers from first- and second-line agents, respectively) presented further MRI activity.

#### Survival analysis for NEDA-3 status and its components

**Fig. 1:**
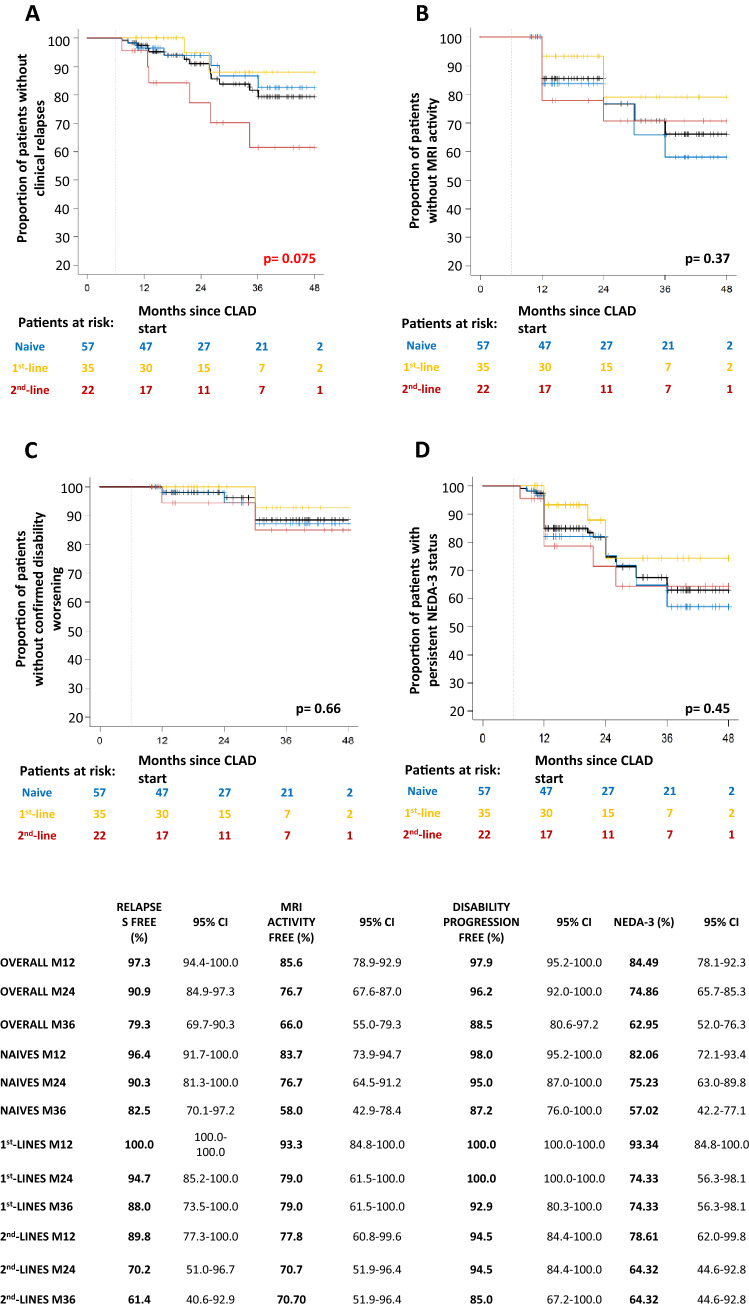
Survival for efficacy outcomes, re-baselined towards months 6, in the whole cohort and in patients stratified according to the last previous DMT before CLAD start, using the Kaplan–Meier method. Proportions of patients without clinical relapses (**A**), MRI activity (**B**), confirmed disability worsening (**C**) and loss of NEDA-3 status (**D**) are shown. Log-rank test *p* values are reported. Number of patients at risk are displayed under each graph. The table below summarizes survival estimates at relevant time points overall and in each group of patients

Figure 1 summarizes the survival for efficacy outcomes, re-baselined towards month 6, in the whole cohort and in patients stratified according to the last DMT before CLAD start, using the Kaplan–Meier method.

At 24 months, 90.9% (95% C.I. 84.9–97.3) of subjects were free from clinical relapses: 90.3% (95% C.I. 81.3–100.0) of naïves, 94.7% (95% C.I. 85.2–100.0) of switchers from first-lines and 70.2% (95% C.I. 51.0–96.7) of switchers from second lines. Patients with MS switching from second-line DMTs tended to display worse survival, compared to naïves and switchers from first-line therapies (*p* = *0.075*).

Globally 76.7% (95% C.I. 67.6–87.0) of patients had no MRI activity at 24 months: 76.7% (95% C.I. 64.5–91.2) of naïves, 79.0% (95% C.I. 61.5–100.0) and 70.7% (95% C.I. 51.9–96.4) of switchers from first- and second-line treatments, respectively.

Overall, 96.2% (95% C.I. 92.0–100.0) of patients showed no disability progression at 24 months: 94.5% (95% C.I. 87.0–100.0) of naïves, 100.0% (95% C.I. 100.0–100.0) of switchers from first-line and 94.5% (95% C.I. 84.4–100.0) of switchers from second-line treatments.

At 24 months since CLAD start, overall 74.9% (95% C.I. 65.7–85.3) were NEDA-3: 75.2% (95% C.I. 63.0–89.8) of naïves, 74.3% (95% C.I. 56.3–98.1) of switchers from first-lines and 64.3% (95% C.I. 44.7–92.8) of switchers from second-line.

No differences among treatment groups were found for MRI activity, confirmed disability progression and NEDA-3 status loss over time (all *p* ≥ *0.37*).

#### Predictors of response

Table 2 summarizes results of univariable and multivariable Cox regression models using time to first relapse, first MRI activity and first loss of NEDA-3 status as outcomes, investigating potential risk factors for suboptimal response to CLAD.

**Table 2:** Univariable and multivariable Cox regression models using time to first relapse, first MRI activity and first loss of NEDA-3 status as outcomes

Variable	Relapse				MRI activity				NEDA-3			
	Univariate analysis		Multivariate analysis		Univariate analysis		Multivariate analysis		Univariate analysis		Multivariate analysis	
	HR (95% CI)	*p* value	HR (95% CI)	*p* value	HR (95% CI)	*p* value	HR (95% CI)	*p* value	HR (95% CI)	*p* value	HR (95% CI)	*p* value
Sex (female vs male)	**4.40 (0.76–25.32)**	**0.097**			2.42 (0.83–7.10)	0.107			2.16 (0.81–5.71)	0.122		
Age at onset	**0.91 (0.84–1.00)**	**0.048**			**0.94 (0.88–0.10)**	**0.043**			**0.95 (0.90–1.01)**	**0.080**		
Age at CLAD start	0.95 (0.89–1.02)	0.157			**0.93 (0.89–0.98)**	**0.009**	**0.95 (0.90–1.00)**	**0.034**	**0.96 (0.91–1.00)**	**0.052**		
EDSS at baseline	**1.55 (1.08–2.23)**	**0.016**	**1.44 (0.96–2.15)**	**0.078**	1.09 (0.75–1.57)	0.652			1.19 (0.860–1.635)	0.299		
Disease duration	1.02 (0.93–1.12)	0.636			0.94 (0.86–1.03)	0.170			0.98 (0.91–1.05)	0.518		
N of relapses 1 year before CLAD start	1.30 (0.66–2.57)	0.448			0.93 (0.56–1.57)	0.793			0.96 (0.59–1.56)	0.857		
N of relapses 2 years before CLAD start	1.05 (0.76–1.45)	0.778			0.95 (0.73–1.23)	0.674			0.94 (0.73–1.20)	0.595		
N of relapses during the first 6 months after CLAD start	1.92 (0.25–14.88)	0.531			1.22 (0.16–9.07)	0.845			0.95 (0.13–7.00)	0.957		
Last previous treatment 1st-line vs naive	**0.60 (0.12–3.01)**	**0.069**	**1.28 (0.23–7.22)**	**0.025**	0.48 (0.16–1.44)	0.424			0.55 (0.20–1.50)	0.485		
2nd line vs naive	**2.68 (0.86–8.31)**		**6.16 (1.49–25.37)**		0.85 (0.31–2.34)				0.98 (0.38–2.51)			
2nd line vs 1st line	**4.43 (0.89–22.01)**		**4.80 (0.93–24.82)**		1.78 (0.47–6.65)				1.79 (0.55–5.89)			
N of previous treatment before CLAD start	1.24 (0.87–1.76)	0.242			0.90 (0.64–1.26)	0.534			1.00 (0.75–1.34)	1.000		
N of baseline T2-hyperintense lesions	1.00 (0.97–1.03)	0.767			1.01 (0.99–1.03)	0.261			1.01 (0.99–1.03)	0.321		
N of baseline Gd + lesions	1.14 (1.04–1.24)	**0.004**	**1.19 (1.07–1.32)**	**< 0.001**	**1.15 (1.06–1.24)**	**< 0.001**	**1.11 (1.03–1.21)**	**0.008**	**1.15 (1.064–1.241)**	**< 0.001**	**1.15 (1.06–1.24)**	**< 0.001**
Presence of active lesions during the first 6 months after CLAD start	1.39 (0.48–4.01)	0.545			0.99 (0.42–2.31)	0.977			1.15 (0.52–2.50)	0.735		

Candidate predictors (*p* < 0.1) at univariable analysis and selected predictors at multivariable analysis are highlighted in bold

Active lesions = new and or/enlarged T2-hyperintense lesions and/or gadolinium-enhancing lesions

*CLAD* cladribine, *EDSS* Expanded Disability Status Scale, *N* number, *GD* + gadolinium-enhancing, *HR* hazard ratio, *CI* confidential interval

A higher baseline EDSS score (hazard ratio (HR) 1.44, 95% confidential interval (CI): 0.96–2.15, *p* = *0.078*), second-line agents as last previous DMT (HR 6.16, 95% CI: 1.49–25.37, *p* = *0.025*) and a higher number of gadolinium-enhancing lesions at baseline MRI (HR 1.19, 95% CI: 1.07–1.32, *p* < *0.001*) independently increased the risk of first clinical relapse during follow-up.

A younger age at CLAD start (HR 0.95, 95% CI: 0.90–1.00, *p* = *0.034*) and a higher number of gadolinium-enhancing lesions at baseline MRI (HR 1.11, 95% CI: 1.03–1.21, *p* = *0.008*) independently predicted first MRI activity during follow-up.

A higher number of brain gadolinium-enhancing lesions at baseline (HR 1.15, 95% CI: 1.06–1.24, *p* < *0.001*) was the only risk factor for first loss of NEDA-3 status during follow-up.

No predictors were found for disability progression (data not shown).

Notably, the presence of relapses and/or MRI activity during the first 6 months after CLAD start did not increase the risk of developing relapses or MRI activity, nor losing NEDA-3 status during follow-up.

LASSO regularized models identified the same subsets of response predictors described above, for each outcome.

#### ARR, MRI activity and EDSS score trend per treatment epoch

**Fig. 2:**
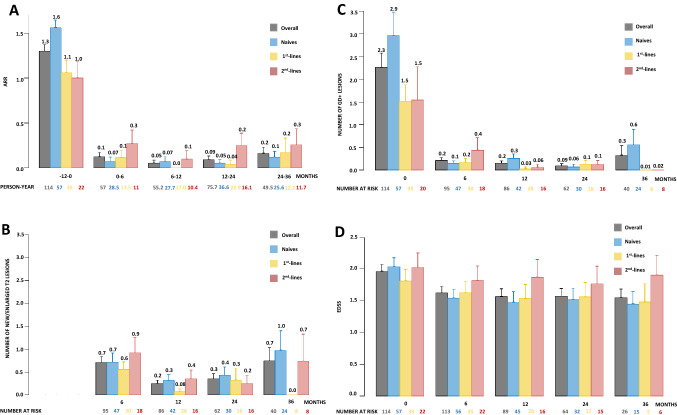
ARR, MRI activity and EDSS score trend over time, overall and according to last previous DMT before CLAD start. Estimates of mean (standard error) ARR (**A**), number of new/enlarging T2 (**B**) and gadolinium-enhancing (**C**) lesions, EDSS score (**D**) per treatment epoch are plotted. Person-year and patients at risk are displayed below each graph

Figure 2 shows ARR, MRI activity and EDSS score trend over time, overall and according to last DMT before CLAD start.

ARR substantially declined in the first 6 months after CLAD start (*p* < *0.001*) and remained stable in the subsequent time points (all *p* ≥ *0.151*). No significant time × group interaction was observed (*p* = *0.311*) (Fig. 2A). Similarly the number of gadolinium-enhancing lesions significantly dropped at month 6 (*p* < *0.001*) and then remained stable (all *p* ≥ *0.160*). Although no significant time × group interaction was observed (*p* = *0.413*), patients switching from second lines showed a residual MRI activity at month 6 (*p* = *0.143)*, significantly reduced at month 12 (*p* = *0.044)* compared to baseline (Fig. 2B).

The number of new/enlarged T2-hyperintense lesions substantially declined between months 6 and 12 (*p* = *0.002*) and then remained stable (all *p* ≥ *0.199*), with no significant time × group interaction (*p* = *0.248*) (Fig. 2C).

We observed a significant reduction in mean EDSS scores in the first 6 months (*p* < *0.001*), followed by a stabilization during follow-up (all *p* ≥ *0.426*). The reduction at month 6 was significant in naïves (*p* < *0.001*), while no time effect was found in switchers from first- (*p* = *0.488*) and second-line (*p* = *0.182*) agents (Fig. 2D). However, no significant time × group interaction was observed (*p* = *0.306*).

12 patients with MS were judged as non-responsive to CLAD and switched to other treatments. One subject started natalizumab after 11 months since CLAD start, before CLAD second course, due to both clinical (1 relapse) and MRI (5 new T2-hyperintense brain lesions, 1 with contrast enhancement) activity. Eleven patients started other DMTs for persistency of MS activity after CLAD second treatment course: 3 patients started teriflunomide, 3 dimethyl fumarate, 2 alemtuzumab, 2 natalizumab and 1 ocrelizumab. Of the 2 patients who switched to alemtuzumab, 1 presented more than 40 T2-hyperintense and 19 gadolinium-enhancing lesions at baseline MRI and reported 2 relapses in the year before CLAD start. The second one previously had received 4 treatments, including fingolimod (last treatment before CLAD start) and cyclophosphamide. Overall, median time to CLAD discontinuation in these 12 patients was 29.6 months since CLAD first administration (IQR 26.7–35.1).

Two patients received an additional course of CLAD due to persistent disease activity after 24 months.

### Cladribine safety profile

#### White blood cell and lymphocyte levels over time

**Fig. 3:**
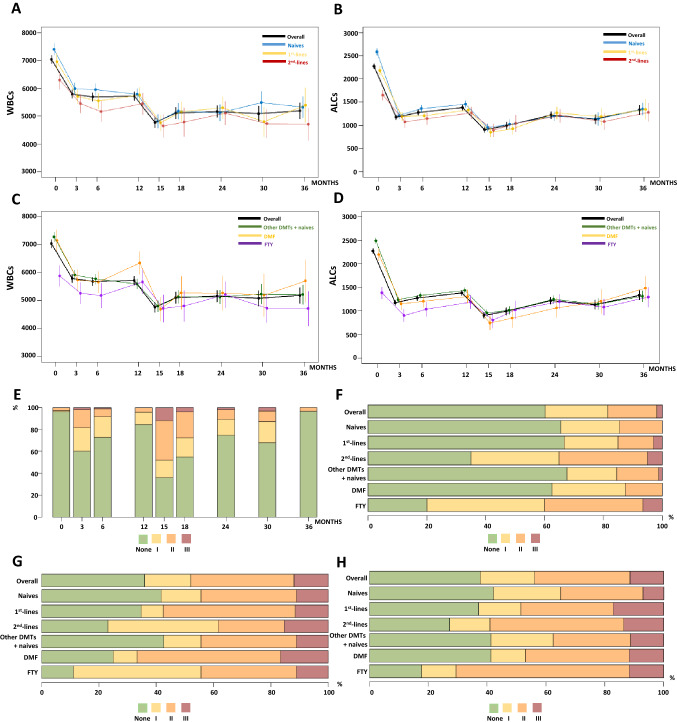
White blood cell counts and absolute lymphocyte levels, along with lymphopenia degree profile, over time, in the whole cohort and according to the different patients’ categories. Estimates of mean (± standard error) white blood cell (**A**) and absolute lymphocyte (**B**) count in the whole cohort, in naives and switchers from first and second lines over time. Estimates obtained evaluating separately patients switching from dimethyl fumarate and fingolimod are also shown (**C** and **D**). The bar plots represent the distribution of lymphopenia severity grade profile in the whole cohort over time (**E**), with a focus at month 3 (**F**), month 15 (**G**) and nadir (**H**), according to all different patient categories considered

Figure 3 represents data on WBCs, ALCs and lymphopenia degrees, over time, in the whole cohort and according to the different patients’ categories.

Overall, our patients largely reflected the well-known pattern of WBC and lymphocyte kinetics following CLAD exposition, with a reduction at months 3 and 15 (all *p* < *0.001*).

Patients switching from second-line agents displayed a lower baseline mean WBC compared to naïves (mean WBC [se]/mm^3^ = 6291[327] and 7394[209], respectively, *p* < *0.005*). More specifically, patients switching from fingolimod had a lower mean WBC (mean WBC [se]/mm^3^ = 5888[371]) at baseline (all *p* ≤ *0.019*).

A significant time × group effect was detected for ALCs between naïves and switchers (*p* < *0.001*), due to a lower mean ALCs at baseline in switchers compared to naïves (mean ACL [se]/mm^3^ = 2583[72] in naives; 2181[93] in switchers from first-lines; 1651[112] in switchers from second lines; all *p* < 0.001). Comparable lymphocyte levels among groups were observed in the subsequent follow-up (all *p* > *0.242*). Compared to naïves and switchers from other DMTs (mean ACL [se]/mm^3^ = 2492[60]*)*, a lower mean ALCs at baseline was found in patients starting CLAD from fingolimod (mean ACL [se]/mm^3^ = 1384[125]; *p* < *0.001*) and dimethyl fumarate (mean ACL [se]/mm^3^ 2194[132], *p* = *0.041*).

At month 3, switchers from fingolimod (mean ACL [se]/mm^3^ = 903[132]) still presented a reduced mean ALC (mean ACL [se]/mm^3^ = 1240[59]; *p* = *0.020*), whereas no differences were observed for patients switching from dimethyl fumarate (all *p* ≥ *0.176*). No other significant differences in mean lymphocyte levels were found in the subsequent follow-up (all *p* ≥ *0.170*).

A higher proportion of patients showed lymphopenia grade ≥ I at month 3 and 15 (Fig. 3E). Notably, no grade IV lymphopenia cases were observed.

At month 3, no differences in the distribution of lymphopenia severity grades were noted between naïves and switchers (*p* = *0.127*), although patients switching from fingolimod presented a higher proportion of grade I and II lymphopenia cases (*p* = *0.016*). No differences were found in the lymphopenia severity distribution both at month 15 and nadir among the different treatment groups (all *p* ≥ *0.195*). Thirteen (11.4%) out of 114 patients presented grade III lymphopenia at their nadir. Prevalence of grade III lymphopenia did not differ among the patient categories considered (all *p* ≥ *0.239*).

Independent predictors of grade III lymphopenia over time were a lower baseline ALC (OR 0.85, 95% CI: 0.74–0.99, *p* = *0.032*, associated to a 100-unit-change in baseline ALC) and a higher number of previous DMTs (OR 1.53, 95% CI: 1.01–2.32, *p* = *0.046*).

#### Tolerability

Overall, 62 (54.4%) patients presented at least one side effect and globally 111 AEs were recorded. At month 3, 44 events were reported: 19 infections, of which 3 SARS-CoV-2 infections, 5 fatigue, 3 skin rash, 8 headache, 6 nausea and/or gastrointestinal disturbances, 3 elevation of liver enzymes and 1 alopecia. At month 15, we recorded 15 AEs: 6 infections, of which 4 SARS-CoV-2 infections, 2 fatigue, 1 skin rash, 1 headache, 3 elevation of liver enzymes and 2 alopecia. No serious and/or clinically significant AEs were noted.

SARS-CoV-2 infections were all asymptomatic and/or mild and spontaneously resolved. No hospitalizations due to COVID-19 were reported.

Grade III lymphopenia did not predict the risk of infections over time (OR 2.41, 95% CI: 0.53–10.93, *p* = 0.253).

## Discussion

Evidence from the real-world experience is of great value in analyzing effectiveness and safety profile of drugs and reflects clinical practice more closely than the selected conditions of clinical trials. Here, we present a monocentric dataset of 114 RRMS patients following CLAD treatment over a mean period of 25 months, evaluating CLAD effectiveness and safety profile and providing clues on predictors of treatment response.

Compared to the pivotal phase III CLARITY clinical trial and its extension, our subjects were overall younger (mean age 33.0 vs 37.9 years), less disabled (mean baseline EDSS 2.0 vs 2.8) and started CLAD earlier (mean disease duration pre CLAD start 3.0 vs 7.9 years) [4].

Our population enrolled a higher proportion of treatment-naïves (50%) compared to recent real-world studies, including the Italian (29.3%) [9], Danish (12.7%) [10] and German (36.0%) [11] cohorts. Our patients were also more active at baseline (ARR 1 year before CLAD start 1.6) compared to the other populations (ARR 1 year before CLAD start ≤ 1.0 in all cohorts) [9–11].

Follow-up post CLAD start was homogeneous and did not differ significantly between naïves and switchers.

Only a minority of patients who presented clinical (6.1%) or MRI (31.6%) activity in the first 6 months since CLAD start developed further disease activity, mainly patients switching from second-line agents, more frequently fingolimod. Notably, the presence of disease activity during the first 6 months did not increase the risk of developing further activity during follow-up, suggesting that a certain degree of initial activity could be tolerated up to the administration of a complete therapeutic cycle. These findings suggest an early onset of action of CLAD and are in line with the those of the MAGNIFY-MS study, which evaluated the onset of action of CLAD on MRI activity during the first 6 months of treatment in highly active RMS patients and demonstrated a significant reduction in active lesion count from month 1 onward compared with the baseline period [12].

Approximately 75% of our patients were NEDA-3 at 24 months since CLAD start, compared to 64% at 22 months in the Italian cohort [9] and 49% at 24 months in the Danish one [10]. NEDA-3 analysis in the Danish study was performed only on 42.2% of the total population. Several factors can contribute to explain the differences between our and the Danish study in terms of NEDA-3, including the fact that we used CLAD earlier (median disease duration pre CLAD start 3.0 vs 8.1 years) and in less-treated patients (percentage of subjects who underwent 2 DMTs before CLAD start 13% vs 25%) [10].

When analyzing subcomponents of NEDA-3, approximately 90% of our patients remained free from relapses at 24 months. In an Australian study on 90 CLAD patients coming from a national MS registry, 65% of subjects was relapse-free at 24 months [13]. Also in the comparison with this cohort, we placed CLAD earlier in the treatment algorithm: mean disease duration pre CLAD start was 3.0 in our vs 13.0 years in the previous study and median number of previous DMTs was 0.5 vs 2.2 [13]. An association between the number of previous DMTs and relapses was found also in a real-world Finnish study on 179 patients under CLAD: patients with two or more previous DMTs had a shorter time to first relapse compared to patients who were treatment-naïve or had used only one previous DMT [14]. In line with our data, a large study on 782 patients under CLAD from the MSBase Registry showed that approximately 85% of subjects were relapse-free at 24 months [15]. In our study, patients switching from second-line DMTs tended to display worse survival rates in terms of relapses, compared to naïves and switchers from first-lines, as previously demonstrated [11, 14]. In a recent study that assessed frequency and severity of relapses in the CLARITY trial, subjects receiving CLAD had a significant lower risk of relapses at month 6, 12 and 24, compared to placebo, confirming a durable effect of CLAD in reducing frequency and severity of relapses [16].

To have some practical pieces of information which could be used for the selection of those patients who might most benefit from the use of CLAD, we also analyzed predictors of first loss of NEDA-3 status over time. The only factor affecting the likelihood of retaining NEDA-3 was the number of brain gadolinium-enhancing lesions at baseline. Patients who were not NEDA-3 at 24 months mainly presented MRI activity and were less likely to lose NEDA-3 due to clinical relapses. Patients with a higher active lesion count at baseline had also an increased risk to develop MRI activity over the follow-up, as previously reported [9]. This result is not surprising, as it is well-known that the presence of gadolinium-enhancing lesions at baseline is a predictor of MRI activity during follow-up [17].

The trend analysis on ARR, MRI activity and EDSS score over time demonstrated that CLAD efficacy occurred relatively rapidly after administration of the first course, with effects on ARR already evident 6 months after CLAD start, as already described [13, 18]. Notably, patients switching to CLAD from second-line treatments showed a residual MRI activity at month 6, significantly reduced at month 12, confirming how naïves and switchers from first-line therapies seem to benefit more from CLAD treatment.

We were not able to perform a specific analysis on patients starting CLAD switching from natalizumab as we only included 4 patients, with a limited follow-up. Real-world evidence on this topic is contradictory, as some authors highlighted how these subjects appeared to be more prone to develop disease activity, due to natalizumab cessation-related reactivation [11], while some others found that this switch was effective and safe [19]. Further evidence on an increasing number of patients with longer follow-up is needed to shed light on this topic. Previous studies demonstrated that CLAD was more efficacious than first-line agents such as interferons, comparable to fingolimod and less efficacious than natalizumab [20–22]. Patients under CLAD had a lower probability of experiencing relapses and a significantly lower ARR than those on interferon, glatiramer acetate and dimethyl fumarate. The likelihood of experiencing relapses was similar to fingolimod and higher than on natalizumab [20–22]. Subjects treated with CLAD displayed longer time-to-treatment discontinuation and were less likely to switch treatment compared to individuals under fingolimod, dimethyl fumarate, or teriflunomide [22]. Our work confirms these findings, even if we did not perform an ad-hoc analysis to directly compare fingolimod, natalizumab and CLAD efficacy.

CLAD was overall well tolerated with a favorable safety profile. WBCs and ALCs in our patients followed the kinetics known from RCTs, with lymphopenia peaking at month 3 and 15 [4, 11, 23]. Incidence of grade III lymphopenia was lower than what reported in clinical trials (11.4% vs 25.0%) and no grade IV lymphopenia cases were recorded, in line with previous findings [14]. Lymphocyte counts mostly recovered before the second-year dose and only 2 subjects needed to postpone retreatment due to persistent lymphopenia. Lower baseline WBCs and ALCs were found for switchers from fingolimod and dimethyl fumarate. At month 3 switchers from fingolimod still presented a reduced ALC compared to all other patients, with no other differences during the subsequent follow-up. The risk of developing grade III/IV lymphopenia was higher in patients with lower ALCs at baseline, consistently with previous studies [9, 24, 25].

We observed no discontinuations due to safety alerts and CLAD was stopped only for efficacy reasons. Infections were the most frequent side effect, none was serious. Grade III lymphopenia did not predict the risk of infections over time. Previous reports highlighted how CLAD was associated with reactivation of herpes zoster [4, 11, 26], but we observed only self-limiting herpes simplex cases and no zoster reactivations.

COVID-19 cases in our population were all mild and self-limiting, in line with previous evidence [27, 28].

Headache was reported by approximately one third of patients. This is in line with data from CLARITY trial and with previously published reports [4, 23]. No new safety alerts and no AEs of special interest were observed in our cohort, as already emerged from a long-term post-authorization safety study comparing patients initiating CLAD (*N* = 606) and fingolimod (*N* = 475) [29].

Our work is not free from limitations. First, this study is observational, therefore data derive from a non-controlled real-world setting and enrolled subjects were not selected in advanced according to established inclusion/exclusion criteria. However, a study-specific spreadsheet for data collection was set up before study initiation to collect a minimal common set of fundamental information for all subjects. Moreover, a longer follow-up and a larger cohort are needed to confirm our findings on long-term outcomes, even though the monocentric design of this study increases the reliability of collected data.

Overall, our real-world study corroborates previous data on effectiveness and safety profile of CLAD, identifying early predictors of response. Our findings support to notion that CLAD is more effective when placed early in the treatment algorithm of patients with RMS.

## Data Availability

The dataset used and analyzed during the current study is available from the corresponding Author on reasonable request.
