# Correction to: Effectiveness and safety profile of cladribine in an Italian real-life cohort of relapsing–remitting multiple sclerosis patients: a monocentric longitudinal observational study

**DOI:** 10.1007/s00415-023-11772-5

**Published:** 2023-05-17

**Authors:** Chiara Zanetta, Maria A. Rocca, Alessandro Meani, Vittorio Martinelli, Laura Ferrè, Lucia Moiola, Massimo Filippi

**Affiliations:** 1 Neurology Unit, IRCCS San Raffaele Scientific Institute, Via Olgettina, 60, 20132 Milan, Italy; 2 Neurorehabilitation Unit, IRCCS San Raffaele Scientific Institute, Milan, Italy; 3 Neuroimaging Research Unit, Division of Neuroscience, IRCCS San Raffaele Scientific Institute, Milan, Italy; 4 Vita-Salute San Raffaele University, Milan, Italy; 5 Neurophysiology Service, IRCCS San Raffaele Scientific Institute, Milan, Italy

**Correction to: Journal of Neurology** 10.1007/s00415-023-11700-7

The original version of this article unfortunately contained a mistake. The several values in Table 1 have shifted up from the correct row. Category of *N* Previous treatments, *N*%” has 4 sub-entries, with which the values should be aligned. And the same is for “Type of Last Previous Treatments, *N*%”, which has 7 sub-entries with which the values should be aligned.

The corrected Table 1 is given in the following page.

**Table 1:** Demographic, clinical and MRI characteristics of multiple sclerosis patients starting cladribine at baseline

	All	Naives	1st-lines	2nd-lines	*p*
Total *N* (female *N*; %)	**114** (**82**; 71.9)	**57** (**42**, 73.7)	**35** (**21**, 60.0)	**22** (**19**, 86.4)	0.102^a^
Age at CLAD start (years), mean (SD)	**33.0** (9.2)	**32.4** (9.9)	**33.3** (9.3)	**34.1** (7.1)	0.747^b^
MS duration pre CLAD start (years), median (IQR)	**3.0** (0.7–8.3)	**0.7** (0.3–1.5)	**7.2** (3.1–11.7)	**8.3** (5.1–12.5)	** < 0.001**^c^
EDSS at CLAD start, median (IQR)	**2.0** (1.5–2.5)	**2.0** (1.5–2.5)	**1.5** (1.0–2.5)	**1.5** (1.0–2.5)	0.159^c^
1 year before ARR, mean (SD)	**1.3** (0.8)	**1.6** (0.6)	**1.1** (0.8)	**1.0** (0.9)	** < 0.001**^c^
2 years before ARR, mean (SD)	**1.2** (0.8)	**1.3** (0.6)	**1.0** (0.7)	**1.3** (1.1)	0.068^c^
Treatment characteristics					** < 0.001**^**c**^
*N* previous treatments, median (IQR)	**0.5** (0.0–2.0)	–	**1.0** (1.0–2.0)	**2.0** (1.0–3.0)	
Category of *N* previous treatments, *N* (%)					
0	**57** (50.0)	**57** (100.0)	–	–	
1	**26** (22.8)	–	**18** (51.4)	**8** (36.4)	
2	**15** (13.2)	–	**10** (28.6)	**5** (22.7)	
> 2	**16** (14.0)	–	**7** (20.0)	**9** (40.9)	
Type of last previous treatments, *N* (%)					
Interferon	**4** (3.5)	–	**4** (11.4)	–	
Glatiramer acetate	**9** (7.9)	–	**9** (25.7)	–	
Teriflunomide	**5** (4.4)	–	**5** (14.3)	–	
Dimethyl fumarate	**17** (14.9)		**17** (48.6)	–	
Fingolimod	**17** (14.9)	–	–	**17** (77.3)	
Natalizumab	**4** (3.5)	–	–	**4** (18.2)	
Ocrelizumab	**1** (0.9)	–	–	**1** (4.5)	
Brain T2-weighted lesion count, median (IQR)	**16.0** (10.0–30.0)	**16.0** (10.0–33.5)	**13.5** (10.0–25.0)	**22.0** (10.0–31.0)	0.538^c^
Brain gadolinium-enhancing lesion count, median (IQR)	**1.0** (0.0–3.0)	**2.0** (1.0–3.0)	**1.0** (0.0–2.0)	**1.0** (0.0–1.5)	**0.019**^**c**^
Follow-up post CLAD start (m), median (IQR)	**25.2** (14.6–38.7)	**24.0** (14.0–39.6)	**22.9** (16.2–34.3)	**30.9** (14.6–43.5)	0.558^c^

*N* number of subjects included in the study, *CLAD* cladribine, *Y* years, *SD* standard deviation, *IQR* interquartile range, *MS* Multiple Sclerosis, *EDSS* Expanded Disability Status Scale, *ARR* Annualized Relapse Rate, *m* months

^a^Fisher’s exact test

^b^linear models

^c^Kruskal–Wallis Test

